# Revisiting overexpression of a heterologous β-glucosidase in *Trichoderma reesei*: fusion expression of the *Neosartorya fischeri Bgl3A* to *cbh1* enhances the overall as well as individual cellulase activities

**DOI:** 10.1186/s12934-016-0520-9

**Published:** 2016-07-11

**Authors:** Xianli Xue, Yilan Wu, Xing Qin, Rui Ma, Huiying Luo, Xiaoyun Su, Bin Yao

**Affiliations:** Key Laboratory for Feed Biotechnology of the Ministry of Agriculture, Feed Research Institute, Chinese Academy of Agricultural Sciences, No. 12 South Zhongguancun Street, Beijing, 100081 People’s Republic of China; College of Biological Sciences and Technology, Beijing Forestry University, Beijing, 100083 People’s Republic of China; College of Life Science and Technology, Huazhong University of Science and Technology, Wuhan, 430074 People’s Republic of China

**Keywords:** *Trichoderma reesei*, Cellulase, β-Glucosidase, DNA assembler, *Neosartorya fischeri*

## Abstract

**Background:**

The filamentous fungus *Trichoderma reesei* has the capacity to secret large amounts of cellulase and is widely used in a variety of industries. However, the *T. reesei* cellulase is weak in β-glucosidase activity, which results in accumulation of cellobiose inhibiting the endo- and exo-cellulases. By expressing an exogenous β-glucosidase gene, the recombinant *T. reesei* cellulase is expected to degrade cellulose into glucose more efficiently.

**Results:**

The thermophilic β-glucosidase *Nf*Bgl3A from *Neosartorya fischeri* is chosen for overexpression in *T. reesei* due to its robust activity. In vitro, the *Pichia pastoris*-expressed *Nf*Bgl3A aided the *T. reesei* cellulase in releasing much more glucose with significantly lower amounts of cellobiose from crystalline cellulose. The *NfBgl3A* gene was hence fused to the *cbh1* structural gene and assembled between the strong *cbh1* promoter and *cbh1* terminator to obtain pRS-*Nf*Bgl3A by using the DNA assembler method. pRS-*Nf*Bgl3A was transformed into the *T. reesei* uridine auxotroph strain TU-6. Six positive transformants showed β-glucosidase activities of 2.3–69.7 U/mL (up to 175-fold higher than that of wild-type). The largely different β-glucosidase activities in the transformants may be ascribed to the gene copy numbers of *NfBgl3A* or its integration loci. The *T. reesei*-expressed *Nf*Bgl3A showed highly similar biochemical properties to that expressed in *P. pastoris*. As expected, overexpression of *NfBgl3A* enhanced the overall cellulase activity of *T. reesei*. The CBHI activity in all transformants increased, possibly due to the extra copies of *cbh1* gene introduced, while the endoglucanase activity in three transformants also largely increased, which was not observed in any other studies overexpressing a β-glucosidase. *Nf*Bgl3A had significant transglycosylation activity, generating sophorose, a potent cellulase inducer, and other oligosaccharides from glucose and cellobiose.

**Conclusions:**

We report herein the successful overexpression of a thermophilic *N. fischeri* β-glucosidase in *T. reesei*. In the same time, the fusion of *NfBgl3A* to the *cbh1* gene introduced extra copies of the cellobiohydrolase 1 gene. As a result, we observed improved β-glucosidase and cellobiohydrolase activity as well as the overall cellulase activity. In addition, the endoglucanase activity also increased in some of the transformants. Our results may shed light on design of more robust *T. reesei* cellulases.

## Background

*Trichoderma reesei* is one of the most important industrial filamentous fungi for its robust secretion of cellulases and is widely used in textile, paper, animal feed, and biofuel industries [1]. *Trichoderma reesei* produces a complete set of extracellular cellulase system including two exo-glucanases (cellobiohydrolases), five endoglucanases, and one β-glucosidase [2], which synergistically act to deconstruct cellulose into simple sugars [3]. The predominant components of the *T. reesei* secretome are cellobiohydrolase I (CBHI) and II (CBHII), which together account for 80–85 % of the overall cellulases [4] and processively degrade crystalline cellulose from the reducing (CBHI) or non-reducing (CBHII) end, generating cellobiose as the main product [5]. Accumulated cellobiose inhibits the activity of endo- and exo-glucanases [6, 7], which can nonetheless be relieved through cellobiose hydrolysis by a β-glucosidase. Therefore, β-glucosidase plays an important role in cellulose degradation [8, 9]. Although *T. reesei* has a complete cellulase system, its β-glucosidase activity is weak and is thought to be a critical bottleneck of the *T. reesei* cellulase efficiency [10].

As an approach to circumvent the intrinsic insufficiency of β-glucosidase activity, expressions of either a heterologous or an endogeneous β-glucosidase have been tried, in which the supplemented β-glucosidases are frequently observed to improve the degrading efficiency of *T. reesei* cellulases [11–13]. With the increased cellulase activity, saccharification of pretreated cornstalk and barley straw is enhanced [13]. Despite a general cellulase activity-enhancing effect, it is noted from these studies that the β-glucosidases of varying origins have largely differing effects on the cellulase performance. This suggests that, by exploring the rich microbial diversity for novel β-glucosidases with potent capability, the *T. reesei* cellulase activity can be significantly improved.

We have previously reported the cloning and biochemical characterization of a new β-glucosidase *Nf*Bgl3A from a thermophilic filamentous fungus *Neosartorya fischeri* [14]. This enzyme is robust, thermostable, and resistant to many chemicals and ions, making it an ideal candidate for supplementing *T. reesei* with the needed β-glucosidase activity. In this study, the *Pichia pastoris*-expressed *Nf*Bgl3A was first added in vitro to the *T. reesei* cellulase and evaluated for its ability to improve the cellulase performance. Then, the *NfBgl3A* β-glucosiase gene was overexpressed in *T. reesei* by fusing with the *cbh1* gene and the overall and individual cellulase activities of the transformants were systematically analyzed.

## Results

### *Nf*Bgl3A enhanced *T. reesei* cellulase in hydrolyzing crystalline cellulose in vitro

**Fig. 1:**
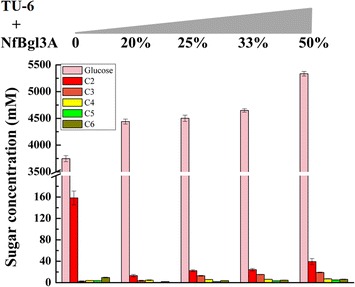
Supplementing with the *P. pastoris*-expressed *Nf*Bgl3A into the *T. reesei* cellulase enhanced release of glucose from crystalline cellulose. *Nf*Bgl3A was expressed in *P. pastoris*, purified, and added to TU-6 cellulase with increasing concentrations (0, 20, 25, 33, and 50 %, w/w). The enzymes were reacted with 20 mg/mL Avicel at 50 °C for 24 h. The released glucose and cellooligosaccharides were analyzed using HPAEC-PAD. C2-C6 represent cellobiose to cellohexaose, respectively

The *P. pastoris*-expressed *Nf*Bgl3A is a robust thermophilic β-glucosidase (optimal temperature: 80 °C) with a specific activity of 2189 ± 1.7 U/mg against *p*-nitrophenol-β-d-glucopyranoside (*p*NPG). At the temperature optimum for *T. reesei* cellulases (50 °C), it still retains significant activity as high as above 50 % [14]. The *T. reesei* TU-6 cellulase (i.e. the fermentation broth of TU-6 on day 6 post Avicel induction, 51.4 μg/mL) alone released mainly glucose (3747.6 ± 57.3 μM) and large amounts of cellobiose (158.4 ± 12.9 μM) with much lower amounts of cellooligosacchrides with degrees of polymerization from three to six (Fig. 1). Increasingly adding the *P. pastoris*-expressed *Nf*Bgl3A into the TU-6 cellulase (20, 25, 33, and 50 % percent, w/w) enhanced release of glucose from 4441.2 ± 46.7 (20 % *Nf*Bgl3A) to 5334.4 ± 42.5 μM (50 % *Nf*Bgl3A), whereas the amounts of cellobiose dramatically decreased to between 13.2 ± 2.1 (20 % *Nf*Bgl3A) and 39.4 ± 5.7 (50 % *Nf*Bgl3A) μM (Fig. 1). The amounts of cellotetraose, cellopentaose, and cellohexaose were nearly unchanged for the reactions supplemented with *Nf*Bgl3A, whereas cellotetraose slightly increased from 2.6 ± 0.9 (no *Nf*Bgl3A) to between 3.7 ± 0.9 (20 % *Nf*Bgl3A) to 19.2 ± 0.7 μM (50 % *Nf*Bgl3A) (Fig. 1). The results clearly demonstrated that *Nf*Bgl3A could enhance the *T. reesei* cellulase activity by decreasing cellobiose accumulation which in turn relieved the inhibition of cellulase.

### Construction of the *NfBgl3A* expressing plasmid by DNA assembler

**Fig. 2:**
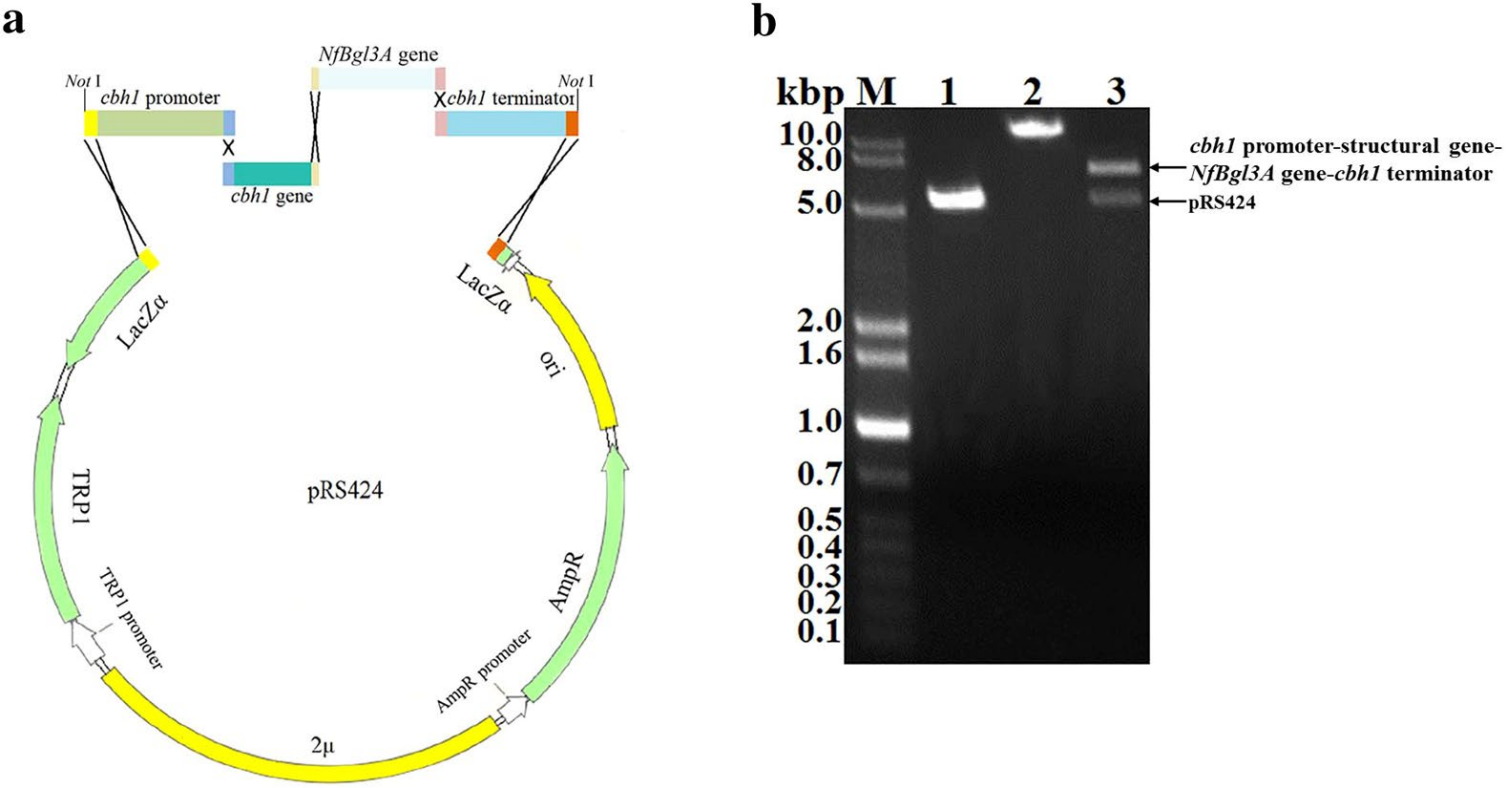
Construction of pRS-*NfBgl3A* using DNA assembler. **a** Schematic diagram showing one-step assembly of pRS-*NfBgl3A.* **b** Restriction digestion analysis of pRS-*NfBgl3A* by *Not*I. *Lane M* DNA molecular weight marker; *lane 1* *Kpn*I-linearized pRS424; *lane 2* undigested pRS-*NfBgl3A*; *lane 3* *Not*I-digested pRS-*NfBgl3A*

Since in vitro supplementing with *Nf*Bgl3A largely enhanced the *T. reesei* cellulase activity, it was expected that extracellular expression and extracellular secretion of *Nf*Bgl3A in *T. reesei* would improve the cellulase efficiency. A recombinant plasmid for overexpressing *NfBgl3A* in *T. reesei* was thus constructed using the fast DNA assembler method, which was initially employed to assemble metabolic pathways in *Saccharomyces cerevisiae.* The DNA assembler method utilizes the yeast highly efficient in vivo homology recombination system [15] and has been applied to construct high throughput gene knockout cassettes in *T. reesei* [16] but has seldom been used in constructing expressing cassettes in *T. reesei.* We used this method to rapidly construct the recombinant plasmid, namely pRS-*Nf*Bgl3A, expressing the new thermophilic β-glucosidase *Nf*Bgl3A using the multicopy plasmid pRS424 as the backbone (Fig. 2a). The expression cassette includes the *cbh1* promoter (1700 bp), the *cbh1* structural gene (1542 bp), the *NfBgl3A* gene (2315 bp), and the *cbh1* terminator (1500 bp). The *cbh1* gene was fused N-terminal to *NfBgl3A* gene, facilitating the latter’s expression and secretion, as well as introducing extra copies of this main *cellobiohydrolase* gene. Note that the introns in the *cbh1* and *NfBgl3A* structural genes were not removed because we believe that *T. reesei* should have the ability to correctly recognize and splice the mRNA precursors of the two fusion genes both originating from filamentous fungi.

The *cbh1* promoter, *cbh1* structural gene, *NfBgl3A* gene, and the *cbh1* terminator were all PCR amplified from corresponding *T. reesei* or *N. fischeri* genomic DNAs and co-transformed with the *Xho*I-linearized pRS424 into *S. cerevisiae* AH109. Long primers with >30 bp overhangs were designed (Table 1), which ensured efficient homologous recombination of the adjacent DNA fragments and correct assembly of the genes into an integrated expressing cassette. A Kex2 endoproteinase recognition site (RDKR, corresponding to the nucleotide sequence CGCGACAAGCGC) [17, 18] was designed between CBHI and *Nf*Bgl3A, which allows automatic cleavage of *Nf*Bgl3A from the fusion protein CBHI–*Nf*Bgl3A during secretion. The integrity of the recombinant plasmid pRS-*Nfbgl3A* was verified by both restriction digest by *Not*I (Fig. 2b) and DNA sequencing (data not shown).

**Table 1:** Primers used in this study

Primer	Sequence (5′–3′)	Usage
Trcbh1pF	TGAGCGCGCGTAATACGACTCACTATAGGGCGAATTGATATCTAGAGTTGTGAAGTCGG	Cloning of *cbh1* promoter
Trcbh1Pr	GCACAATACGACTCCGGCGCTGGCCGATGCGCAGTCCGCGGTTGACTATT	Cloning of *cbh1* promoter
TrCBH1F	CCCAATAGTCAACCGCGGACTGCGCATCATGTATCGGAAGTTGGCCGTCATCTCGGCC	Cloning of *cbh1* structural gene
TrCBH1R	CACCAGAACCGTAGCGCTTGTCGCGCTCGAGCAGGCACTGAGAGTAGTAAGGGTTCAGG	Cloning of *cbh1* structural gene
NfBgl3AF	TTACTACTCTCAGTGCCTGCTCGAGCGCGACAAGCGCTACGGTTCTGGTGGCAGCAACTGGGATC	Cloning of *NfBgl3A* gene
NfBgl3AR	TACGGGCTCACCAAGAATCTACCGGTGCGTCAGGCTTTCGCCACGGAGCTTCACCATCCGC GACGGACCCTGAAAGAG	Cloning of *NfBgl3A* gene
Trcbh1tF	ACCACCACCACCACCACTAACTCGAGAGCTCCGTGGCGAAAGCCTGACGCACCGG	Cloning of *cbh1* terminator
Trcbh1tR	CCACCGCGGTGGCGGCCGCTCTAGAACTAGTGGATCCCCCGGGCTGCAGGGCGGCCGCAACA CTTCGGTGGAGGTGTCGAGTACG	Cloning of *cbh1* terminator
Yz-Trchb1F	GTTCGGACCCATTGGCAGCACCGG	Screening of positive transformants
Yz-NfBgl3AR	AAGC GGATACCTAGCGGCGAGTCCTG	Screening of positive transformants
q-NfBgl3AF	AATGGGCGCTGAGGCGAAGGGAC	qRT-PCR
q-NfBgl3AR	TGAGAGCGGTGGTATCCACG	qRT-PCR
q-tef1α-F	ACCAAGGCTGGCAAGTTC	qRT-PCR
q-tef1α-R	GACACCAGTCTCGATACG	qRT-PCR

### The *NfBgl3A* transformants have largely different β-glucosidase activity

**Fig. 3:**
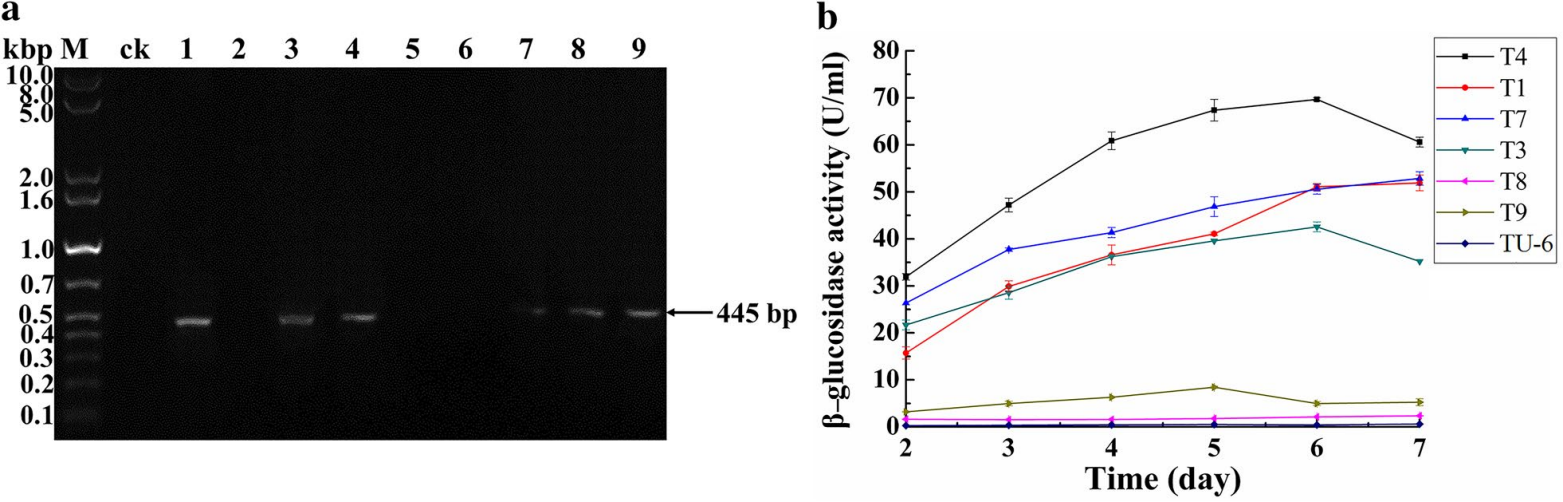
Transformation and expression of *NfBgl3A* in *T. reesei.* **a** Determination of *NfBgl3A* integration in the genome of *T. reesei* transformants by PCR using the primers Yz-Trchb1F/Yz-*Nf*Bgl3AR. The expected size is 445 bp, corresponding to a fragment spanning the joint region of *cbh1* structural gene and *NfBgl3A*. *Lane M* DNA molecular mass marker; *lane* T1–T9, the transformants of T1–T9. **b** The β-glucosidase activities in the fermentation broth of TU-6 and six transformants

pRS-*Nf*Bgl3A was co-transformed with pSKpyr4, which provides uridine prototrophy, into *T. reesei* TU-6. After regeneration of the protoplasts, nine transformants (T1 to T9) were randomly picked out. The integration of *NfBgl3A* expression cassette in the genome of TU-6 was verified by PCR (Fig. 3a). Among these transformants, six strains, that were T1, T3, T4, T7, T8, and T9, showed the brightest band corresponding to the expected size (445 bp) and were consequently selected for further analyses and comparison with their parent strain TU-6.

TU-6 had low β-glucosidase activity over the whole induction phase (0.26–0.58 U/mL) as reported previously [8] (Fig. 3b). T4 had the highest β-glucosidase activity over the fermentation period (day 2 to 7 post cellulose induction), while T1, T3, and T7 showed similar β-glucosidase activity to each other but much higher than T8 and T9 (Fig. 3b). The β-glucosidase activity of T4, T1, T3, and T7 remarkably increased from day 2 to 6 post induction, whereas those of T8, T9, and TU-6 did not increase significantly (Fig. 3b). The highest β-glucosidase activity (69.7 ± 0.4 U/mL) for T4 was at day 6 post Avicel induction, which was approximately 175-fold of TU-6 (Fig. 3b). The maximum β-glucosidase activities of T1 (51.9 ± 0.2 U/mL), T7 (52.9 ± 1.4 U/mL), and T3 (42.5 ± 1.3 U/mL) were 130-, 132- and 106-fold of TU-6, respectively.

### Correlation of the *NfBgl3A* copy numbers with the β-glucosidase activity of the transformants

**Fig. 4:**
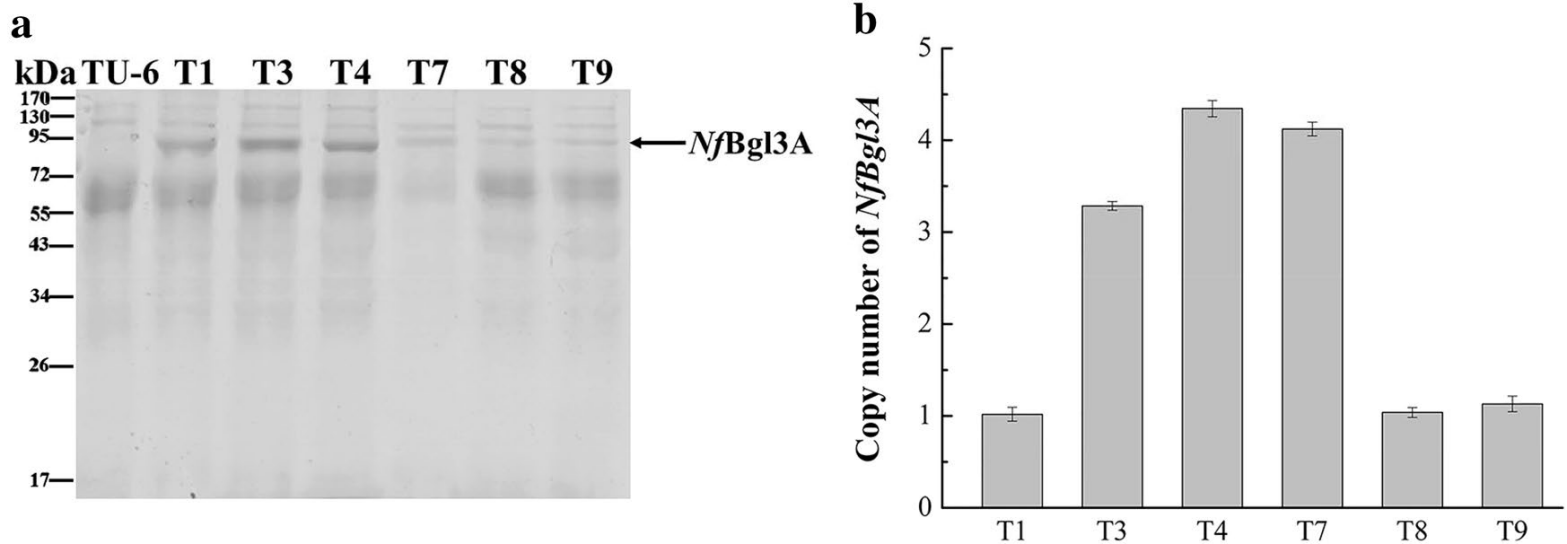
SDS-PAGE analysis of *NfBgl3A* expression and its copy numbers in the transformants. **a** SDS-PAGE analysis of the crude enzymes of TU-6 and the *NfBgl3A* transformants. The culture supernatants on day 5 post induction were used. **b** Determination of the *NfBgl3A* copy numbers by qPCR analysis

The largely differing β-glucosidase activity in the *NfBgl3A* transformants suggested that *Nf*Bgl3A might be expressed with varying degrees. Indeed, the SDS-PAGE analysis of the culture supernatants indicated that more *Nf*Bgl3A was secreted by transformants T1, T3, and T4 (Fig. 4a). Since the copy number is one of the critical factors affecting heterologous gene expression [19], we determined the copy number of *NfBgl3A* in the transformants.

The qPCR analysis of the transformants indicated that the copy numbers of the *NfBgl3A* gene were different in these strains. T4 and T7 had four copies of the *NfBgl3A* gene, while T3 had three copies (Fig. 4b). The high copy numbers of these strains were in accordance with high level β-glucosidase expression in *T. reesei*. Although T1 had only one copy of *NfBgl3A* gene, its β-glucosidase activity was similar to that of T7 and even higher than T3, whereas T8 and T9 had only one gene copy of *NfBgl3A* but much lower β-glucosidase activity. This suggested that the integration locus other than the gene copy numbers might also affect the expression of the β-glucosidase.

### The β-glucosidase *Nf*Bgl3A expressed in *T. reesei* has similar biochemical property to that expressed in *P. pastoris*

**Fig. 5:**
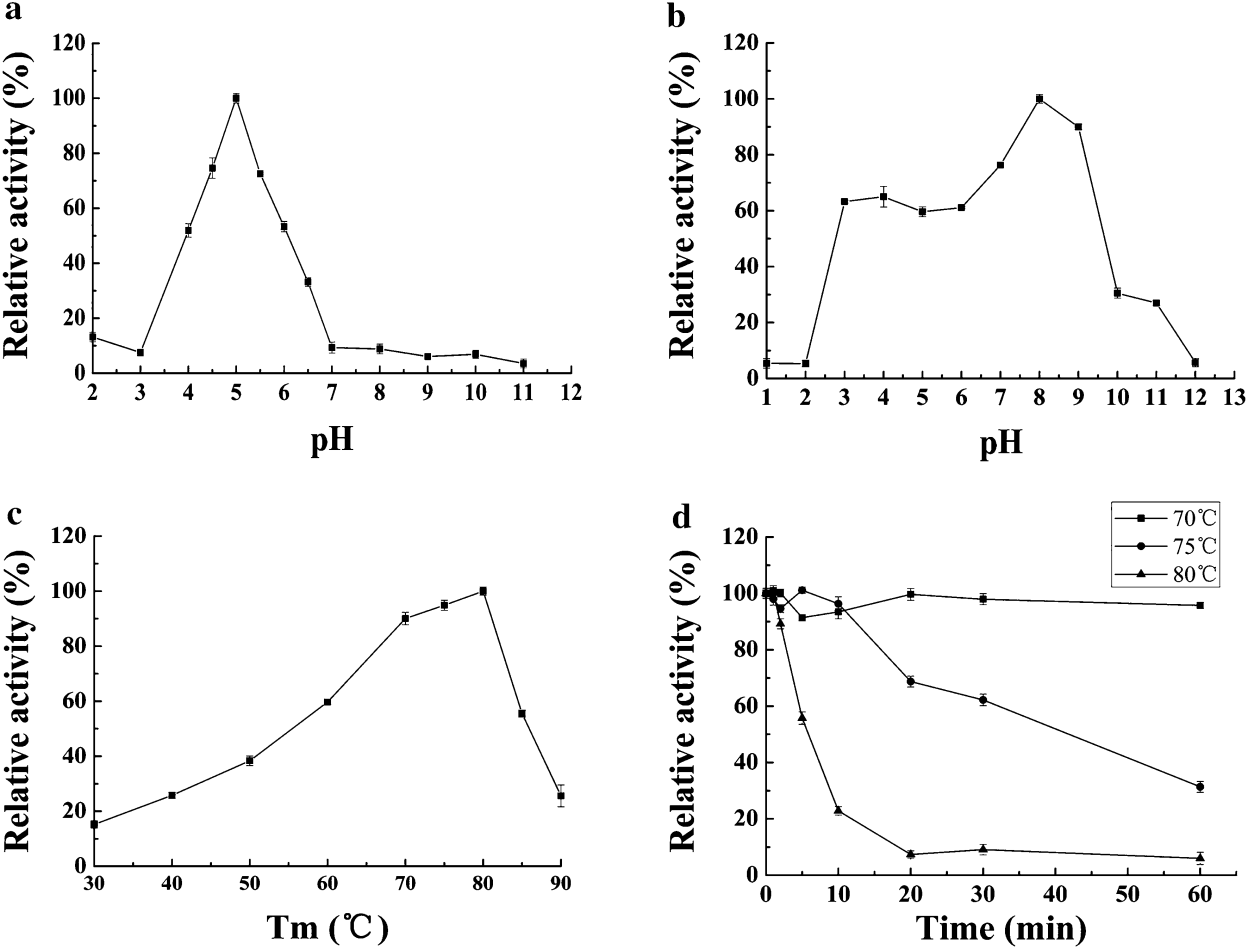
Biochemical characterization of *Nf*Bgl3A expressed in *T. reesei*. **a** Effect of pH on β-glucosidase activity. The enzyme assays were performed at 80 °C for 10 min. **b** pH stability. The crude *Nf*Bgl3A was pre-incubated without substrate at 37 °C for 1 h and then subjected to assay of the residual activity under standard conditions (pH 5.0, 80 °C, 10 min). **c** Effect of temperature on *Nf*Bgl3A activity. The assays were carried out at pH 5.0 for 10 min. **d** Thermostability. The crude *Nf*Bgl3A was pre-incubated in the McIlvaine buffer (pH 5.0) at 70, 75, or 80 °C and the residual β-glucosidase activities were measured

The fermentation broth of T4 on day 6 post induction with the highest β-glucosidase activity was used as crude *Nf*Bgl3A (tr*Nf*Bgl3A). The crude enzyme showed similar biochemical properties to that expressed in *P. pastoris* [14]. tr*Nf*Bgl3A had an optimum pH of 5.0 and temperature of 80 °C (Fig. 5a, c), which were the same as that expressed in *P. pastoris.* tr*Nf*Bgl3A had approximately 80 % activity at pH 4.5–5.5. Additionally, tr*Nf*Bgl3A was stable over a broad range of pH, retaining >65 % of the activity after incubation at pH 3.0–9.0 for 1 h (Fig. 5b). tr*Nf*Bgl3A was thermostable at 70 °C for 1 h (Fig. 5d).

### Improved overall and individual cellulase activities in the pRS-NfBgl3A transformed *T. reesei*

**Fig. 6:**
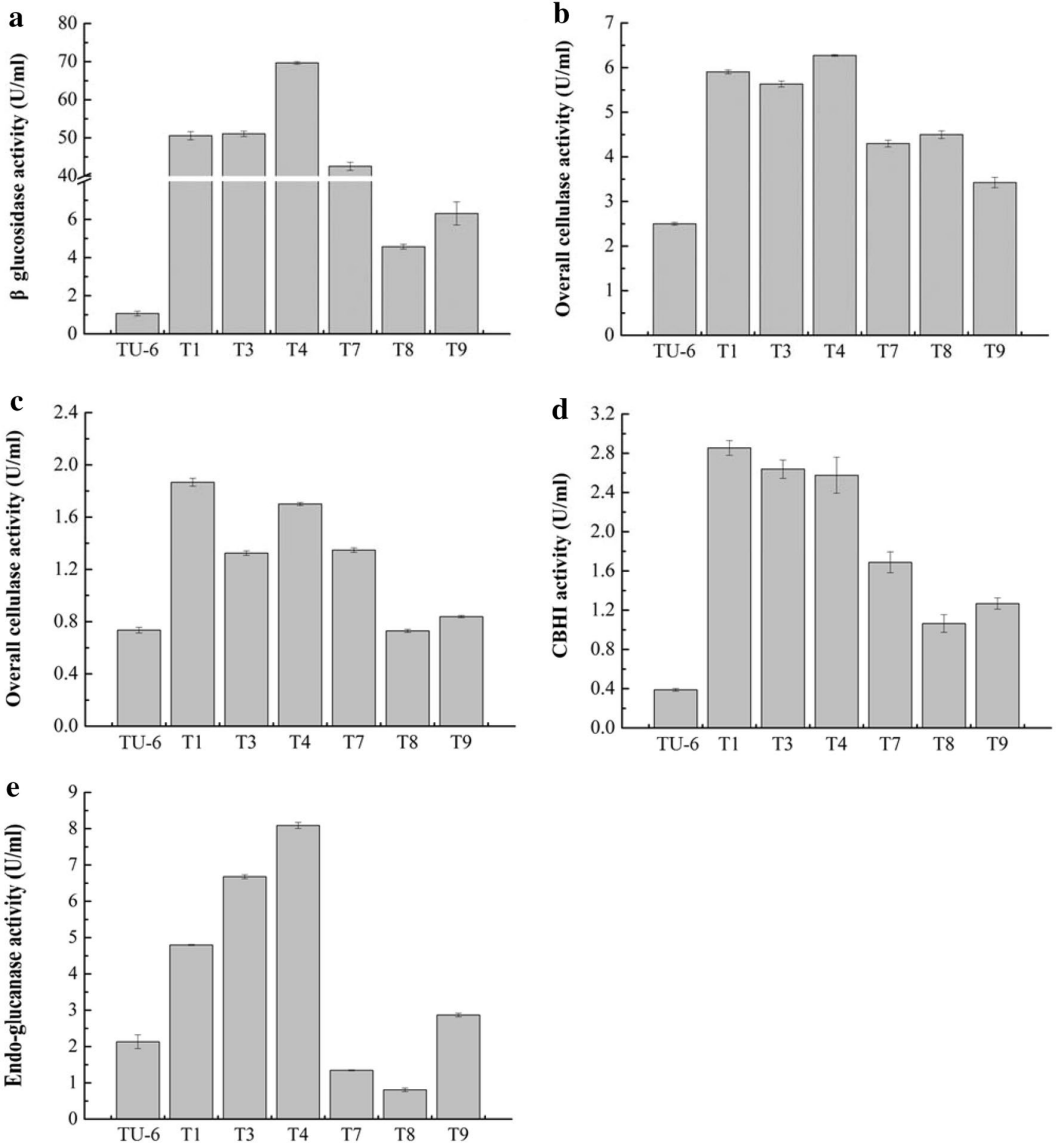
Overall and individual cellulase activities of TU-6 and the transformants. **a** The β-glucosidase activity. **b**, **c** The overall cellulase activities on Avicel (**b**) and filter paper (**c**), respectively. **d** CBHI activity as determined using MUC as the substrate. **e** The endo-glucanase activity using CMC-Na as the substrate

Since the highest β-glucosidase activity was detected on day 6 post Avicel induction (Fig. 3b), the fermentation broth of TU-6 and its *NfBgl3A* transformants at this time was subjected to protein concentration determination and cellulase assays using a variety of substrates. The protein concentration of the fermentation broth of TU-6 was 51.4 μg/mL. For T1, T3, T4, T7, T8, and T9, the values were 84.0, 109.2, 217.3, 78.8, 55.4, and 63.8 μg/mL, respectively. As mentioned before, T4 had the highest β-glucosidase activity, followed by T1, T3, and T7 (Figs. 3b, 6a). T8 and T9 had lower activities, which were nevertheless still higher than that of TU-6. Most of the pRS-NfBgl3A transformed *T. reesei* had improved overall cellulase activity against Avicel (Fig. 6b) and filter paper (Fig. 6c). Higher cellulase activity was commonly (T4, T1, T3, and T7) but not always (compare T7 and T8) associated with better β-glucosidase activity (Fig. 6b, c). While the elevated protein concentration in the transformants seemed to generally have a positive effect on the overall cellulase activity, a clearly proportional relationship could not be identified. Nevertheless, the higher the β-glucosidase activity was, the higher the protein concentration in the fermentation broth of the transformants (T1, T3, T4, and T7).

The CBHI activity increased in all transformants, with T1 (2.9 ± 0.1 U/mL), T3 (2.6 ± 0.1 U/mL), and T4 (2.6 ± 0.2 U/mL) being the highest, followed by T7 (1.7 ± 0.1 U/mL), T9 (1.3 ± 0.1 U/mL) and T8 (1.1 ± 0.1 U/mL) (Fig. 6d). TU-6 had a much lower CBHI activity of 0.4 ± 0.0 U/mL. This can be explained by extra copies of the *cbh1* gene introduced in the transformants. It appeared that the endo-glucanase activity (CMCase activity, Fig. 6e) was affected in some transformants. While TU-6 had an endo-glucanase activity of 2.1 ± 0.2 U/mL, T4, T3, and T1 had much higher activities of 8.1 ± 0.1, 6.7 ± 0.2, and 4.8 ± 0.2 U/mL, respectively. T9 had a slightly increased activity of 2.9 ± 0.1 U/mL. Conversely, the endo-glucanase activities decreased for T7 (1.3 ± 0.0 U/mL) and T8 (0.8 ± 0.0 U/mL).

**Fig. 7:**
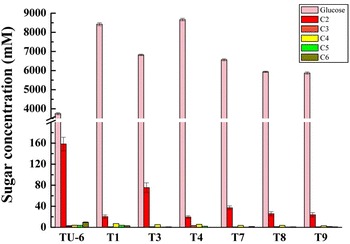
Hydrolysis of Avicel by TU-6 and its transformants as analyzed by HPAEC-PAD. The fermentation broth of strain TU-6 and its transformants harboring *Nfbgl3A* on day 6 post the induction was used to hydrolyze 20 mg/mL of Avicel. The reactions were carried out at 50 °C for 24 h. The released glucose and cellooligosaccharides were analyzed using HPAEC-PAD. *C2*–*C6* represent cellobiose to cellohexaose, respectively

In accordance with the in vitro *Nf*Bgl3A-supplementation experiment, extracellular expression of *Nf*Bgl3A enhanced release of glucose by all transformants from 5873.2 ± 62.1 (T9) to 8666.9 ± 67.0 μM (T4) compared with 3747.6 ± 57.3 μM by TU-6, as analyzed by HPAEC-PAD (Fig. 7). Note that the concentrations of released glucose by all transformants, particularly by T1 and T4, were significantly higher than those released by TU-6 supplemented with different amounts of *P. pastoris*-expressed *Nf*Bgl3A. Higher amounts of released glucose are basically positively related to stronger β-glucosidase activities (T4 and T1). For all transformants, the concentrations of cellotetraose, cellopentaose, and cellohexaose were similar to those of TU-6. No increase of cellotriose was observed for any of the transformants, which may be ascribed to the low ratio of *Nf*Bgl3A against the total protein: the densitometry analysis by Image-Pro plus 6.0 software (Media Cybernetics Company, America) revealed that *Nf*Bgl3A expressed in T4 did not exceed 20 % of the total protein (data not shown). This complied with the in vivo supplementation experiments, in which only supplementation with >20 % *Nf*Bgl3A increased release of cellotriose (Fig. 1). Cellobiose in all transformants decreased to 19.7 ± 2.4 to 35.5 ± 9.1 μM, which were much lower than that released by TU-6 (158.4 ± 12.9 μM) (Fig. 7).

### *Nf*Bgl3A displays significant transglycosylation ability in vitro

**Fig. 8:**
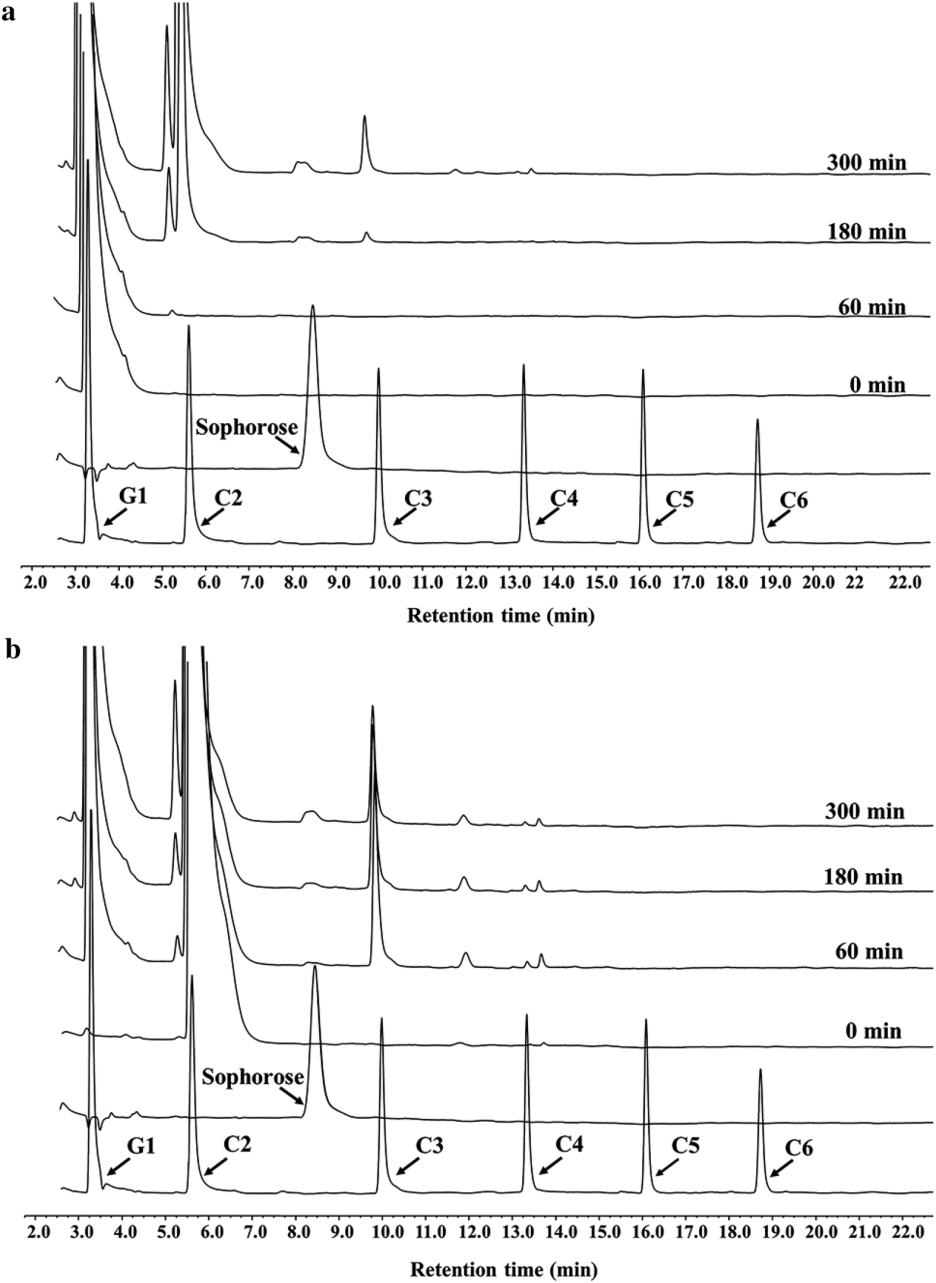
*Nf*Bgl3A displayed transglycosylation activity. *Nf*Bgl3A was incubated with 150 mM of glucose (**a**) or 250 mM of cellobiose (**b**) at pH 5.0 and 70 °C. Samples were taken out periodically and analyzed by HPAEC-PAD. *G1* glucose; *C2*–*C6* cellobiose to cellohexaose

The *P. pastoris*-expressed *Nf*Bgl3A was tested for transglycosylation activity using glucose (150 mM) and cellobiose (250 mM) as the substrates, respectively. In a time-course analysis, *Nf*Bgl3A produced mainly cellobiose from glucose at the end of the reaction (300 min); however, significant amounts of sophorose and cellotriose also began to accumulate after 180 min (Fig. 8a). When cellobiose was used as the substrate, sophorose and cellotriose were also observed (Fig. 8b).

## Discussion

It is well known that the *T. reesei* cellulase is weak in β-glucosidase activity, which may be ascribed to the relatively low expression level [20] as well as to the low specific activity (0.58 ± 0.032 U/mL) [9] of the extracellular Bgl1 (GenBank accession number: AAA18473.1). Inefficient β-glucosidase activity results into accumulation of cellobiose, which inhibits cellobiohydrolase and endoglucanase activities. Relieving such an inhibition by cellobiose requires more potent β-glucosidase in *T. reesei* cellulase. This can be accomplished by supplementing with commercially available β-glucosidases such as that from *Aspergillus niger* [21], or alternatively, by overexpressing either an endogenous or a heterologous β-glucosidase gene in *T. reesei*. Successful overexpressions include the *T. reesei* BGLI [4, 6] and BGLII [5], the *P. decumbens* Bgl1 [7], and the *Periconia* sp. β-glucosidase [8]. *Nf*Bgl3A is a thermophilic β-glucosidase from *N. fischeri* with a specific activity of 2189 ± 1.7 U/mg, which makes it one of the most robust β-glucosidases that have ever been characterized. In addition, although it has an optimal temperature of 80 °C, it still retains over 50 % activity at 50 °C, the temperature commonly used for *T. reesei* cellulase. Its optimal pH is 5.0, identical to that of *T. reesei* cellulase. These merits make *Nf*Bgl3A an attractive candidate β-glucosidase that will work efficiently with the *T. reesei* cellulase. Importantly, we demonstrated that the biochemical characteristics of *Nf*Bgl3A expressed in *T. reesei* recapitulated that expressed in *P. pastori*.

While the parent strain TU-6 displayed low level of β-glucosidase activity, some of the *NfBgl3A* transformants (T4, T1, T7, and T3) had very high β-glucosidase activity. The varied β-glucosidase activities in different transformants may arise from the copy numbers of *NfBgl3A* integrated into the genome or are from the genomic loci of *NfBgl3A* integration, which has been commonly observed for filamentous fungi [14]. The β-glucosidase activities in *NfBgl3A*-overexpressing transformants were much higher than those of transformants expressing the *T. reesei*-BGLI [11], *P. decumbens*-BGL1 [12], and *Periconia* sp.-BGLI [13]. The transformants of *P. decumbens*-Bgl3A had a β-glucosidase activity of as high as 34.31 ± 3.13 U/mL, which is nevertheless still lower than the *NfBgl3A* transformants T4. Since the β-glucosidases appear to be similarly well expressed in *T. reesei* in these studies, the high specific activity of *Nf*Bgl3A is regarded to account for the higher β-glucosidase activity of the transformants in current study.

As expected, the overall cellulase activity was improved in most transformants to different extents. The maximal folds of activity improvement were 2.5-fold for T4 on Avicel and 2.4-fold for T1 on filter paper. Note that increased CBHI activity was also observed for all *NfBgl3A*-overexpressing transformants. This was not observed in previous studies, in which the cellobiohydrolases were only moderately changed or unchanged [11–13]. This is not unexpected since the *NfBgl3A* gene was fused to *cbh1* gene in our study, which introduced additional copies of *cbh1* in the transformants and is in contrast with the previous studies, in which the β-glucosidase genes were not fused to *cbh1*. Therefore, the extra copies of *cbh1* gene should at least partially account for the increased cellobiohydrolase activity. The endoglucanase activities of the transformants, as reflected by the activities against CMC, were either largely upgraded (for T1, T3, and T4), slightly up-regulated (for T9) or down-regulated (for T7 and T8). This is also in contrast to previous β-glucosidase overexpressing experiments, in which the endoglucanase activity of the transformants were down-regulated or kept unchanged [11–13]. The folds of overall cellulase activity on crystalline cellulose (Avicel and filter paper) are neither simple addition nor multiplication of individual activities. This could be explained by the complexity of the canonical cellulase system model, which has cellobiohydrolase, endogluanase, and β-glucosidase [22]. In brief, the overall cellulase activity is a representation of the synergy of these different but complementary types of enzymes, which is related to both the enzyme components’ concentrations, ratios, and the reaction conditions such as substrate characters and concentrations.

The activities of endoglucanases in some transformants were largely altered, which were not observed in former β-glucosidase overexpression studies, either. Integration of *NfBgl3A* might have disrupted expression of a certain gene(s), which in turn affected expression of cellulases. In addition, a β-glucosidase may have transglycosylation activity that allows the enzyme to generate either cellooligosaccharides or oligosaccharides with altered glycosidic linkages (such as sorphorose) [23] acting as inducers of cellulase. Indeed, we found that *Nf*Bgl3A have significant transglycosylation activities on both glucose and cellobiose, generating sophorose as one of the products (Fig. 7a, b). It is well-known that sophorose acts as a strong inducer of cellulase expression in *T. reesei*. We found addition of minor amounts of sophorose (36.5 μM) in an Avicel-inducing TU-6 culture significantly enhanced the endo-glucanase activity from 2.8 ± 0.2 to 4.0 ± 0.4 U/mL at day 5 post Avicel induction (data not shown). Therefore, it is possible that sophorose (or other oligosaccharides) generated by *Nf*Bgl3A might be involved in altering the expression profile of *T. reesei.* One may think that the growth condition of *T. reeesi* (28–30 °C, and comparably lower concentrations of glucose and cellobiose in the medium than those in the transglycosylation experiment) is not optimal for *Nf*Bgl3A’s transglycosylation activity; however, note that *Nf*Bgl3A is a highly active enzyme and retains significant activity at 30 °C (Fig. 5c). In addition, sophorose is a potent inducer of cellulase; therefore, even minor amounts of sophorose might result in more effective induction of cellulase in presence of Avicel cellulose in those transformants. In spite of these pieces of evidence, nevertheless, more experiments are required in the future to better understand the mechanisms underlying the findings. Our study revisited overexpression of a heterologous β-glucosidase gene in *T. reesei* by fusing it to the major cellobiohydrolase gene *cbh1* and discovered that the overall as well as the individual cellulase activities in *T. reesei* were enhanced.

## Conclusions

The weak β-glucosidase activity is a bottleneck of the *T. reesei* cellulase in biomass deconstruction. In this study, the *NfBgl3A* gene coding for a highly active and thermotolerant β-glucosidase (*Nf*Bgl3A) from *N. fischeri* was successfully overexpressed in *T. reesei* TU-6 by fusing C-terminal to the major cellobiohydrolase gene *cbh1*. Compared with in vitro addition of *Nf*Bgl3A to the *T. reesei* cellulase, overexpressing *Nf*Bgl3A in *T. reesei* with this special design not only enhanced the glucose yield more significantly, but also improved the cellulose-degrading performance of the major cellulase components, including the cellobiohydrolase and endoglucanase. *Nf*Bgl3A had transglycosylation activity and generated sophorose, a potent inducer of *T. reesei* cellulase, as a product from both glucose and cellobiose. This was hypothesized to be one of the reasons accounting for increased endoglucanase activity in some of the tranformants.

## Methods

### Microbial strains and culture conditions

The *Escherichia coli* Trans I-T1 (Transgen, Beijing, China) strain was used throughout this study for plasmid propagation. The *S. cerevisiae* AH109 (Clontech, San Francisco, CA) auxotrophic for adenine, histidine, leucine, methionine, tryptophan and uracil was used as the host for DNA assembler [15]. The *E. coli* was cultured in Luria-Bertani (LB) medium with appropriate concentrations of ampicillin at 37 °C when needed. The *S. cerevisiae* was cultivated in yeast peptone dextrose medium with adenine (YPDA) at 30 °C. The thermophilic filamentous fungus *Neosartorya fischeri* strain P1 (CGMCC 3.15369) [24] is stored in our lab. For extraction of its genomic DNA, *N. fischeri* was grown in the potato dextrose medium at 45 °C. The *T. reesei* TU-6 (ATCC MYA-256) strain, which is a uridine auxotrophic strain derived from QM9414 (ATCC 26921) [25], was a kindly gift from Prof. Zhiyang Dong (Institute of Microbiology, Chinese Academy of Science) and used as a host for overexpression of *NfBgl3A*. The TU-6 and its transformants were grown on potato dextrose agar (PDA) containing 10 mM of uridine for sporulation [26] at 28 °C. The *T. reesei* strains were grown in a minimal medium ((NH_4_)_2_SO_4_, 5.0 g/L; KH_2_PO_4_, 15 g/L; MgSO_4_, 0.6 g/L; CaCl_2_, 0.6 g/L; FeSO_4_·7H_2_O, 0.005 g/L; MnSO_4_·H_2_O, 0.0016 g/L; ZnSO_4_·7H_2_O, 0.0014 g/L; CoCl_2_, 0.002 g/L) supplemented with appropriate carbon sources (2 % glucose for growth; or 2 % Avicel for cellulase induction) [27].

### Supplementing with *Nf*Bgl3A in *T. reesei* cellulase

*Nf*Bgl3A was recombinantly produced in *P. pastoris* and purified according to the method described in [14]. Increasing amounts of *Nf*Bgl3A (0, 20, 25, 33, and 50 %, w/w) were added in vitro to the TU-6 cellulase on day 6 post Avicel induction and incubated with 20 mg/mL of Avicel in a total volume of 1.0 mL. The reactions were carried out at 50 °C for 24 h. After the reaction, the enzymes were removed from the reaction system by using a Nanosep centrifugal 3 K device (Pall, New York, NY) and the released glucose and celloligosaccharides were analyzed using HPAEC-PAD (high performance anion exchange chromatography with pulsed amperometric detection).

For HPAEC-PAD analysis, 500 μL of appropriately diluted hydrolysates were applied to a Thermo Scientific Dionex ICS-5000 (Dionex Corporation, Sunnyvale, CA) high-performance liquid chromatography (HPLC) instrument equipped with a CarboPac PA100 guard column (4 by 50 mm) and an analytical column (4 by 250 mm). The flow rate was 1 mL/min and the analysis was carried out at 22 °C. The samples were resolved in a mobile phase gradient from 0 to 100 mM NaOH using glucose and cellooligosaccharides (cellobiose to cellohexaose) as standards.

### Construction of the *NfBgl3A*-expressing plasmid by DNA assembler

The DNA assembler method [15], which utilizes the highly efficient in vivo homologous recombination machinery of the baker’s yeast, was chosen to rapidly construct the *NfBgl3A*-expressing plasmid. The pRS424 plasmid (New England Biolabs, Beverly, MA), a shuttle vector for yeast and *E. coli*, was used as the backbone plasmid. The plasmid was linearized by restriction digestion with *Xho*I (TaKaRa, Dalian, China). The *cbh1* promoter, structural gene, and the terminator were amplified from the genomic DNA of *T. reesei* TU-6 (primers listed in Table 1). The *NfBgl3A* gene was amplified from the genomic DNA of *N. fischeri* P1.

The *Xho*I-linearized pRS424 plasmid and the four DNA fragments, including *cbh1* promoter, *cbh1* structural gene, *cbh1* terminator, and the *NfBgl3A* gene, were mixed and transformed into AH109 by a LiAc-mediated chemical transformation method using a Yeastmaker Yeast Transformation System 2 kit (Clontech, San Francisco, CA). Briefly, 5 μL each of the linearized pRS424 and the four DNA fragments were mixed with equal volumes of Herring Sperm DNA (Promega, Madison, WI) and then added into 100 μL of *S. cerevisiae* competent cells, followed by adding 500 μL of PEG/LiAc. The mixture was incubated at 30 °C for 30 min. Thereafter, the mixture was added with 20 μL of DMSO, incubated at 42 °C for 15 min, and then centrifuged. The supernatant was discarded and the cells were re-suspended with 1 mL of YPDA medium and recovered by incubating at 30 °C for 90 min. Aliquots of 50 μL were spread on synthetic dropout nutrient medium without tryptophan (SD-Trp) plates for selection of positive transformants. The colonies were initially screened for assembly of the DNA fragments by PCR with the gene specific primers of Yz-Trchb1F/Yz-*Nf*Bgl3AR (Table 1) spanning the joint regions of *cbh1* promoter and the *NfBgl3A* gene. The positive colonies were picked out, inoculated into the SD-Trp liquid medium, and cultured at 30 °C with rigorous shaking. The yeast plasmid was extracted using a Yeast Plasmid Miniprep kit (TIANGEN, Beijing, China) and chemically transformed into the *E. coli* Trans I-T1 competent cells for propagation. Since a *Not*I restriction site was added to both the *cbh1* promoter and terminator (Fig. 2a), the recombinant plasmids were verified for assembly of the five DNA fragments by *Not*I restriction digestion followed by agarose gel electrophoresis. The recombinant pRS424 plasmid with the correct assembly of the *cbh1* promoter-structural gene, *NfBgl3A* gene, and the *cbh1* terminator was named pRS-*Nf*Bgl3A.

### Transformantion of *T. reesei*

A pSKpyr4 plasmid, which was constructed by inserting the *pyr4* expressing cassette from *T. reesei* into the pBluescript SK(+) [28], was used for complementation of the uridine auxotrophy [29]. The *T. reesei* TU-6 was co-transformed with the *Not*I-linearized pRS-*Nf*Bgl3A and *Cla*I/*Eco*RI-digested pSKpyr4 plasmids using the PEG-mediated protoplast transformantion method essentially the same as that described previously [27]. Briefly, *T. reesei* Tu-6 was cultured in 100 mL of minimal medium supplemented with 2 % glucose at 28 °C for 16 h. The mycelia were collected by filtration through 8-layers of gauze. Then 10 mg/mL of lysing enzyme (Sigma-Aldrich, St. Louis, MO) and 1 mg/mL of Cellulase ONOZUKA R-10 were simultaneously added to the mycelia and the mixture was incubated at 30 °C with gentle shaking until large amounts of protoplasts were released. Five μg each of pSKpyr4 and pRS-*Nf*Bgl3A were used to co-transform the TU-6 protoplasts and the plates were incubated at 28 °C for 5–7 days until transformants could be clearly visualized. The successful transformation of the *NfBgl3A*-expressing cassette into TU-6 was determined by PCR using the primers Yz-Trchb1F/Yz-*Nf*Bgl3AR (Table 1).

### Induction of *Nf*Bgl3A expression in *T. reesei*

Six *NfBgl3A* positive transformants (namely T1, T3, T4, T7, T8, and T9) were used for flask fermentation. Spores (10^7^) were inoculated into 50 mL of liquid minimal medium supplemented with 2 % glucose and cultured at 28 °C with agitation at 200 rpm for 48 h. The mycelia were filtered by passing the culture through a 200-mesh sifter and washed twice with the minimal medium without any carbon source. Two grams of the mycelia were added to 100 mL of the minimal medium supplemented with 2 % of Avicel. The culture was continued at 28 °C to induce production of the cellulases. From day 2 to 7 post inoculation, 2 mL of the culture were periodically collected for assay of the cellulase activities.

### Assay of β-glucosidase and endo/exo-glucanase activities

For assay of the β-glucosidase activity, *p*NPG was used as the substrate. The reaction consisted of 400 μL of 1.25 mM *p*NPG dissolved in McIlvaine buffer (200 mM Na_2_HPO_4_, 100 mM citric acid, pH 5.0) and 100 μL of appropriately diluted enzymes. The mixture was incubated at 50 °C for 10 min and terminated by adding 1.5 mL of 1 M Na_2_CO_3_. After cooling down to room temperature, the optical density at 405 nm (OD_405_) was measured. One unit of β-glucosidase activity was defined as the amount of enzyme that released 1 μmol of *p*-nitrophenol in one minute.

The cellulase activity can be presented as the overall cellulase activity or divided into endo-glucanase, exo-glucanase (or cellobiohydrolase), and β-glucosidase activity. For the overall activity, filter paper and Avicel were used as the substrate in the assays. For assay of the endo-glucanase activity, sodium carboxymethyl cellulose (CMC-Na) was used as the substrate. The reactions contained 100 μL of appropriately diluted enzymes and 900 μL of 1 % (w/v) substrate (for Avicel and CMC-Na) or one strip of Whatman No.1 filter paper (6 × 1 cm) in the McIlvaine buffer (pH 5.0) and were incubated at 50 °C for 1 h. Then 1.5 mL of the DNS reagent was added to the mixture and boiled for 5 min to terminate the reaction [30]. The OD_540_ of the reactions was measured. One unit of the overall cellulase or endo-glucanase activity was defined as the amount of enzyme that released 1 μmol of reducing sugar per min under the assay conditions.

To determine the activity of CBHI, the dominant cellulases produced by *T. reesei*, 4-methylumbelifery-β-d-cellobioside (MUC) was used as the substrate [31]. Neither CBHII nor EGII react with the MUC substrate [32]. The standard reaction system contained 200 μL of 2 mM MUC with 25 μL of 1 M glucose, with or without 50 mM cellobiose, and 25 μL of appropriately diluted enzymes in the McIlvaine buffer (pH 5.0). The reaction was incubated at 50 °C for 20 min, followed by addition of 250 μL of 1 M Na_2_CO_3_. In the reactions with glucose only, high concentration of glucose inhibited the action of β-glucosidase on MUC. Therefore, MU was released by CBHI and endoglucanase I (EGI). In the reactions with glucose and cellobiose, cellobiose inhibited the activity of CBHI. Therefore, the activity of CBHI could be calculated based on the difference of MUC released in the two reactions. One unit of CBHI activity was defined as the amount of enzyme that released 1 μmol of MU per min under the assay conditions.

### Determining copy numbers by qPCR

To determine the copy numbers of the integrated *NfBgl3A* gene in the transformants, the fungal genomic DNAs were isolated using a Fungal DNA extraction kit (TianGen, Beijing, China) and were used as the template for quantitative PCR (qPCR). The qPCR method was essentially the same as that described by Solomon [33]. The *Tef1α* (translation elongation factor 1-alpha) gene was used to represent a single copy gene [34]. The qPCR was performed with the SYBR Green Real-time PCR Master Mix (TOYOBO, Osaka, Japan) in a CFX96 Real Time PCR Detection System (Bio-Rad, Hercules, CA), using 96-well white PCR plate sealed with ABsolute qPCR seals (Thermo, Waltham, MA). The primers used for the genes were listed in Table 1. A melt curve analysis was performed at the end of each run from 55 to 95 °C with a ramp speed of 0.5 °C to ensure specific sequences amplification of all primers and only one melting temperature on the melting curve. To determine the efficiencies between 90–110 % of all reactions, the genomic DNA samples were diluted serially to construct standard curves and a temperature gradient was used in RT-PCR. The 25 μL reactions each contained 1 μL of diluted DNA as the template, 12.5 μL of 2× SYBR Green Real time PCR Master Mix, 1 μL each of the primers (0.4 M), and 9.5 μL of H_2_O. Equal volume of water instead of DNA was used as a negative control. The qPCR was performed as follows: 94 °C for 30 s, 40 cycles of 94 °C for 30 s, 60 °C for 30 s, and 72 °C for 30 s. The data were analyzed using Bio-Rad iQ5 2.1 Standard Edition Optical System Software. The products of qPCR were also analyzed by electrophoresis on 2.0 % agarose gels.

### Hydrolysis of crystalline cellulose Avicel by TU-6 and its transformants

The fermentation broth of TU-6 and the transformants on day 6 post induction was used to hydrolyze Avicel. The reaction mixture contained 20 mg/mL of Avicel and appropriately diluted broth. The total volume of all these reaction was 1.0 mL. The reactions were carried out at 50 °C for 24 h. After the reaction, the enzymes were removed from the reaction system by using a Nanosep centrifugal 3K device before HPAEC-PAD analysis.

### Biochemical characterization of the β-glucosidase *Nf*Bgl3A expressed in *T. reesei*

To biochemically characterize the recombinant β-glucosidase *Nf*Bgl3A expressed in *T. reesei*, the crude enzyme (i.e. the fermentation broth) of T4 on Day 6 post Aivcel induction was used for analyses. Using *p*NPG as the substrate, the pH optimum of the *T. reesei*-expressed *Nf*Bgl3A (tr*Nf*Bgl3A) was determined in a series of buffers including glycine–HCl (pH 1.0–3.0), McIlvaine buffer (pH 3.0–8.0), Tris–HCl (pH 7.0–9.0), and glycine–NaOH (pH 9.0–12.0) all with a concentration of 100 mM. To determine the optimal temperature of tr*Nf*Bgl3A, the reaction was performed at temperatures ranging from 30 to 90 °C at pH 5.0. For pH stability analysis, tr*Nf*Bgl3A was pre-incubated in a wide range of pH from 1.0 to 12.0 without substrate at 37 °C for 1 h. Then the residual activities were measured under optimal conditions (pH 5.0 and 80 °C). The thermostability of tr*Nf*Bgl3A was determined by measuring the residual β-glucosidase activity after pre-incubating the crude enzyme at 70, 75 or 80 °C for different periods of time.

### Transglycosylation of *Nf*Bgl3A

To determine if *Nf*Bgl3A has transglycosylation capacity, the purified *Nf*Bgl3A recombinantly produced in *P. pastoris* was incubated with 150 mM of glucose or 250 mM of cellobiose at pH 5.0 and 70 °C for a serial of time (0, 60, 180, and 300 min). The total volume of all these reaction was 1.0 mL, which included 900 μL of glucose or cellobiose and 100 μL of appropriately diluted *Nf*Bgl3A. After the reaction, the enzyme was removed from the reaction system by a Nanosep centrifugal 3K device before HPAEC-PAD analysis. Glucose, sophorose, and cellooligosaccharides (cellobiose to cellohexaose) were used as standards.

## Abbreviations

GH: glycoside hydrolase
DNS: 3,5-dinitrosalicylic acid
HPAEC-PAD: high-performance anion exchange chromatography with pulsed amperometric detector
